# Relating Crystal Structure to Surface Properties: A Study on Quercetin Solid Forms

**DOI:** 10.1021/acs.cgd.2c00707

**Published:** 2022-09-19

**Authors:** Panayiotis Klitou, Ian Rosbottom, Vikram Karde, Jerry Y.Y. Heng, Elena Simone

**Affiliations:** † School of Food Science and Nutrition, Food Colloids and Bioprocessing Group, University of Leeds, Woodhouse Ln., Woodhouse, LeedsLS2 9JT, United Kingdom; ‡ Department of Chemical Engineering, Imperial College London, Imperial College Rd, South Kensington, LondonSW7 2AZ, United Kingdom; § Department of Applied Science and Technology, Politecnico di Torino, Corso Duca degli Abruzzi, 24, 10129TorinoTO, Italy

## Abstract

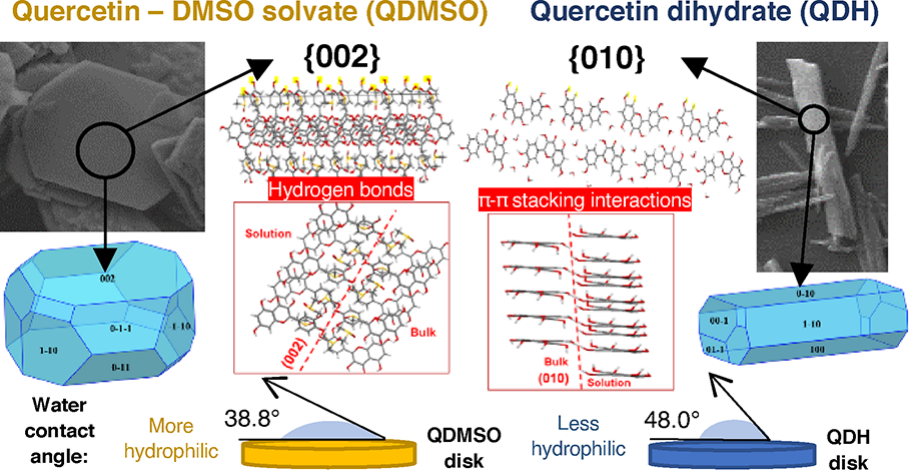

The surface energy and surface chemistry of a crystal are of great importance when designing particles for a specific application, as these will impact both downstream manufacturing processes as well as final product quality. In this work, the surface properties of two different quercetin solvates (quercetin dihydrate and quercetin DMSO solvate) were studied using molecular (synthonic) modeling and experimental techniques, including inverse gas chromatography (IGC) and contact angle measurements, to establish a relationship between crystal structure and surface properties. The attachment energy model was used to predict morphologies and calculate surface properties through the study of their growth synthons. The modeling results confirmed the surface chemistry anisotropy for the two forms. For quercetin dihydrate, the {010} facets were found to grow mainly by nonpolar offset quercetin–quercetin stacking interactions, thus being hydrophobic, while the {100} facets were expected to be hydrophilic, growing by a polar quercetin–water hydrogen bond. For QDMSO, the dominant facet {002} grows by a strong polar quercetin–quercetin hydrogen bonding interaction, while the second most dominant facet {011} grows by nonpolar π–π stacking interactions. Water contact angle measurements and IGC confirmed a greater overall surface hydrophilicity for QDMSO compared to QDH and demonstrated surface energy heterogeneity for both structures. This work shows how synthonic modeling can help in the prediction of the surface nature of crystalline particles and guide the choice of parameters that will determine the optimal crystal form and final morphology for targeted surface properties, for example, the choice of crystallization conditions, choice of solvent, or presence of additives or impurities, which can direct the crystallization of a specific crystal form or crystal shape.

## Abstract 2

The surface properties of two different quercetin solvates were studied using molecular (synthonic) modeling and experimental techniques to establish a relationship between crystal structure and surface properties. For quercetin dihydrate, the dominant {010} facets grow mainly by nonpolar offset quercetin−quercetin stacking interactions, thus being hydrophobic, while for QDMSO, the dominant facet {002} grows by a strong polar quercetin−quercetin hydrogen bonding interaction, thus being more hydrophilic. Water contact angle measurements and IGC verified the modeling calculations and demonstrated surface energy heterogeneity for both structures.

## Introduction

The surface energy and its distribution along the different crystalline facets play an important role in both downstream processing and product performance.^1−3^ For example, these properties have been found to significantly influence the performance of dry powder inhalers, powder mixing, and tablet hardness.^4−6^ Crystal surface energy can influence the particle agglomeration, wetting phenomena, and behavior of particle dispersions in liquids.^5^ Furthermore, unfavorable surface properties can disturb the operating conditions of downstream operations and affect product stability during storage.^7,8^ It is therefore vital to have a good knowledge of the surface properties of crystalline powders and to understand how such properties are affected by the crystal structure (e.g., different polymorphs or solvates). Such knowledge can enable the design of particles with targeted surface properties via either the choice of a specific crystal structure or manipulation of the particle morphology to maximize the area of the facets with the desired surface chemistry (exploiting the intrinsic anisotropy of faceted crystals).

Molecular modeling can be used to predict crystal morphology and to provide a vital insight into the facet specific chemical nature of crystalline materials, as well as their surface anisotropy.^9−13^ Synthonic engineering tools, such as the HABIT software, allow morphological and surface chemistry predictions through the calculation of the ″extrinsic synthons″, the synthons that are unsaturated (broken) at the crystal facets due to their most energetically favorable termination.^14−19^ These extrinsic synthons are important as they impact the physical and chemical properties of the crystals, for example, the crystal growth rate of specific facets, particle shape and aspect ratio, tendency to agglomerate, etc.^20,21^ An understanding of the facet specific extrinsic synthons can aid in the design of crystals with optimal surface properties.

Although the use of molecular modeling and crystal engineering approaches to attempt to rationalize macroscopic particle properties (e.g., thermal, mechanical, solubility) is becoming more common, these are still not widely used to investigate surface properties. This is likely due to the difficulty in experimentally measuring particle surface properties, as well as in computationally describing crystalline surfaces, particularly for complex structures such as solvates or cocrystals.^22,23^

The experimental determination of bulk surface properties of powders includes contact angle measurements and inverse gas chromatography (IGC).^4^ Finite dilution inverse gas chromatography (FD-IGC) has been demonstrated as a practical technique for measuring surface energy in a range of probe molecule surface coverages. Facet specific measurements include probe force and atomic force microscopy as well as X-ray photoelectron spectroscopy; however, these are more challenging techniques that require large crystals for satisfactory results.^3,5,6,24−26^

In our previous work, it was demonstrated that quercetin, a bioflavonoid substance widely used in the food and nutraceutical industries, can exist as an anhydrous pure form or as different solvated structures, including two types of hydrates and a DMSO solvate, which possess different physiochemical properties.^27−31^ Our previous study focused on highlighting crystallographic differences among structures, relating them to bulk properties including thermal stability and tendency to hydration. The work presented here instead analyzes the facet specific surface chemistry of two selected quercetin crystal structures (the dihydrate and the DMSO solvate) via a holistic approach comprising experiments and synthonic modeling. The extrinsic synthons and surface energies of the two structures are calculated and related to the facet measured particle polarity. The role of the solvent molecules on the facet chemical characteristics is discussed. The modeling calculations are compared to experimental surface properties measurements, including inverse gas chromatography and water contact angle measurements. The work aims to provide a complete and comprehensive study of the differences in surface properties between the different solid forms of quercetin. Understanding the surface chemistry of crystalline solids and controlling it via crystal engineering approaches (e.g., choice of appropriate crystal structure, control of crystal morphology) can lead to a rational and quicker particulate product and process design.^32−35^

## Experimental Section

### Materials

Quercetin dihydrate with a purity of 97% was obtained from Alfa Aesar (Port of Heysham Industrial Park, Lancashire, England). Dimethyl sulfoxide (DMSO) solvent was purchased from Fisher Scientific (Bishop Meadow Road, Loughborough, England), and ethanol solvent (99.98%) was purchased from VWR Chemicals. Water purified by treatment with a Milli-Q apparatus was used.

### Recrystallization of Quercetin Dihydrate (QDH)

A 200 g solution of 90% (w/w) ethanol and 10% water solvent with a quercetin concentration of 0.01 g/g was prepared at 20 °C. The quercetin dihydrate was recrystallized by adding water as the antisolvent until the final solvent mixture was 45% (w/w) ethanol/55% water. The first 100 g of water was added at a rate of 400 mL/h using a Cole-Parmer syringe infusion pump. At the end of the first addition, 0.3 g of QDH seeds (from the bottle) was added to the solution, and a further 100 g of water was added to the solution at a rate of 50 mL/h. The temperature was controlled using a Huber Ministat 230 thermoregulator and a PT100 temperature probe connected to a 500 mL jacketed vessel. The crystals were then vacuum filtered using disposable paper filters.

### Crystallization of Quercetin-DMSO Solvate (QDMSO)

A 100 g solution of 60% (w/w) DMSO and 40% water solvent with a quercetin concentration of 0.05 g/g was prepared via heating to 50 °C to ensure the complete dissolution of the solid material. Such solution was then subjected to cooling at a rate of −0.3 °C/min to a temperature of 10 °C. The temperature was then cycled from 10 to 14 °C at a cooling/heating rate of ±0.5 °C/min for 24 h to promote the growth of the crystals and Ostwald ripening. The temperature was controlled using a Huber Ministat 230 thermoregulator and a PT100 probe connected to a 100 mL jacketed vessel. The crystals were then vacuum filtered using disposable paper filters.

### Inverse Gas Chromatography

The QDMSO and QDH crystals obtained as previously described were studied for their surface energy heterogeneity using inverse gas chromatography (iGC-SEA, SMS, UK). IGC data have been shown to give a very robust and reproducible estimation of the dispersive component of the surface energy at different surface coverages of alkane probe molecules.^5,36^ Due to the difference in the specific surface areas of the two samples, different amounts of samples were used for the analysis. About 25 mg of the QDH crystals of size approximately 30 μm in length and 115 mg of the QDMSO crystals of size approximately 300 μm in length were packed into a silanized glass column (internal diameter = 4 mm) and plugged with silanized glass wool on both the ends. A jolting voltameter (Surface Measurement Systems, London, UK) was used to provide mechanical tapping to the sample to remove the voids in the packed sample bed. The packed sample column was placed into the column oven and conditioned at the analysis temperature of 30 °C and 10% relative humidity (RH) for 2 h under 10 mL/min carrier gas (helium) flow rate prior to each measurement. Helium was used as a carrier gas at a flow rate of 10 mL/min, and methane was used as a reference gas to determine the dead volume. The RH was kept at 10% to avoid the dehydration of the QDH sample, which would occur at a lower RH. Both forms were previously shown to be stable under the temperature and relative humidity conditions used during the IGC analysis;^31^ as further confirmation of this hypothesis, Brunauer–Emmett–Teller specific surface area (BET-SSA) measurements were carried out for both QDH and QDMSO at different RH levels (up to 50%). Results of this analysis are reported in the Supporting Information (Tables S1 and S2 and Figure S1) and indicate almost negligible interference of the water molecules with the crystals analyzed. The IGC analysis was carried out in the finite dilution range using a series of n-alkane probes like nonane, octane, heptane, and hexane to determine the dispersive interactions.

### Contact Angle Measurements and Wettability

The water contact angle measurements were carried out at 25 °C using an OCA25 drop-shape tensiometer (DataPhysics Instruments, Germany) fitted with a microsyringe and a high-speed camera. Compressed discs of the QDH and QDMSO samples were prepared by placing 0.3 g of QDH or 0.6 g of QDMSO between the plates of a hydraulic bench press (Clarke, UK) using a 1.54 cm diameter die under a weight of 6 t for 30 s. Static contact angles were measured using the sessile drop method. Water droplets (3 μL) were produced using a straight needle of 0.52 mm outer diameter to form a sessile drop onto the compressed particle disc surfaces. A video camera was used to record the droplet behavior. The droplet contour was fitted using the SCA V.20 software, and the contact angles between the compressed disc and the water droplet were measured.

This technique measures a single parameter over all sites of a compressed powder surface, and the angle measured is an average depending on the relative area of the different facets present on the surface of the compressed disc. Facet specific water contact angle measurements were not possible due to the fact that crystals of suitable size for QDH or QDMSO could not be obtained. All measurements were repeated at least six times to ensure consistency of measurements using three different discs for each material.

### Powder X-ray Diffraction (PXRD)

PXRD was used to confirm the quercetin solid form and identify the morphologically dominant facets in the crystals analyzed. This was estimated by a comparison of the experimental and predicted diffractogram from the crystal structure, where the reflection that was significantly enhanced in the experiment as compared to the theoretical was assumed to be the dominant plane. PXRD patterns were collected on a Panalytical X’Pert PRO that was set up in Bragg–Brentano mode, using Cu Kα radiation (λ = 1.54184 Å), in a scan between 5 and 50° in 2θ with a step size of 0.032° and time per step of 25 s.

### Scanning Electron Microscopy (SEM)

The crystal morphologies of the two quercetin forms were imaged using SEM. The dry samples were imaged using a Carl Zeiss EVO MA15 scanning electron microscope. Samples were arranged on Leit tabs attached to SEM specimen stubs, and an iridium coating was applied before measurement.

## Computational Procedures

The crystallographic information files (.cif) for the two quercetin structures used in the analysis were obtained from the Cambridge Structural Database (CDS): quercetin dihydrate (REFCODE: FEFBEX) and quercetin-DMSO solvate (REFCODE: VUVHOM).^31,37^

Computational analysis was performed using Materials Studio 2017, HABIT98, and Mercury CSD 2020.3.^14,38,39^ The structures were minimized using the Forcite module in Materials Studio 2017 using methodologies described in previous publications.^21,30,38^ The files were exported as .car files (Cartesian coordinates) and converted to fractional coordinates, and then fractional charges were calculated using the AM1 method within MOPAC.^40^ The synthonic analysis was carried out using the HABIT98 software, which takes in structural information to construct a series of unit cells in three dimensions and calculates the pairwise intermolecular interaction between a molecule in the origin unit cell and all the other molecules within a fixed radius of 30 Å from the central molecule.^14,21,41^ The calculation of intermolecular interaction energies was performed using the Momany and Dreiding II force fields.^16,18^ The ranking of the intermolecular interactions by strength was outputted using the DEBUG-1 function. All visualizations of molecular and crystal packing were carried out in Mercury CSD 2020.3.^39^

### Morphology and Surface Chemistry Calculations

The most likely growth slices and BFDH morphologies were calculated using the BFDH morphology calculation feature in Mercury CSD 2020.3 based on the fact that the facets with the largest interplanar spacing (*d*_hkl_) are likely to be morphologically important.^31,39,41,42^ For the slices with the largest interplanar spacing, the lattice energy, *E*_latt_, was partitioned into a slice energy, *E*_sl_, and attachment energy, *E*_att_, according to eq 1:^41,43,44^
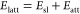
1where the slice energy, *E*_sl_, is the summation of all the interactions between a central molecule and all other molecules within a growth slice of thickness *d*_hkl_ and the attachment energy, *E*_att_, is the summation of all the interactions between the central molecule and molecules outside the growth slice. The attachment energy can be taken to be proportional to the growth rate of that facet according to eq 2:
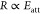
2

The relative attachment energies of each surface were expressed as center to facet distances and then used to determine the external morphology based on the ″attachment energy rule″. Furthermore, the surface anisotropy factor was calculated to provide a measure as to how satisfied the possible intermolecular interactions of a molecule at a growing surface are when compared to those of a molecule within the bulk according to eq 3:
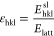
3

## Results

### Experimental and Predicted Morphology of Quercetin Crystals

**Figure 1:**
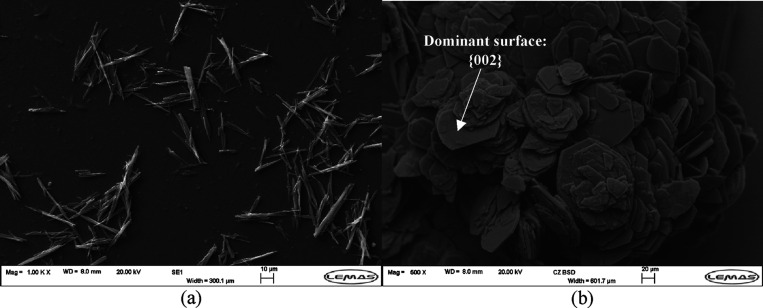
SEM images for (a) QDH grown from an ethanol–water solvent and (b) QDMSO grown from a DMSO–water solvent.

**Figure 2:**
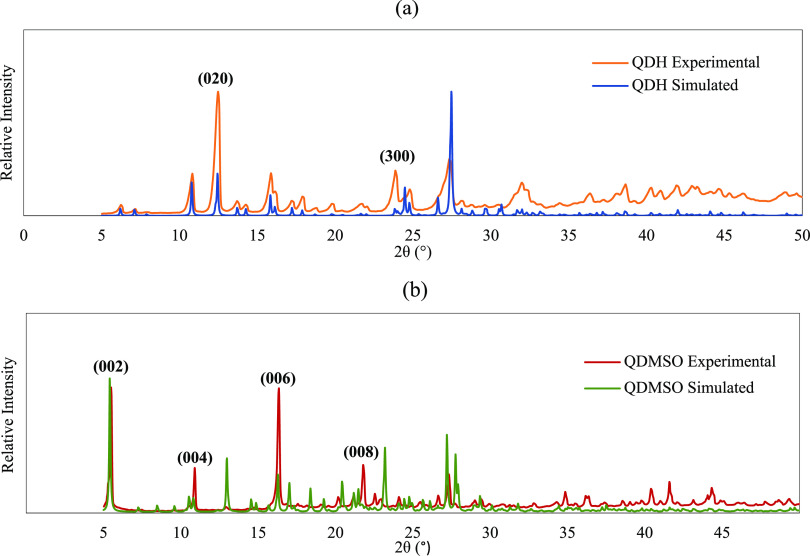
PXRD patterns for simulated and experimental crystal structures of (a) QDH and (b) QDMSO.

Crystals of QDH and QDMSO, grown from an ethanol–water solvent and a DMSO–water solvent, respectively, are shown in Figure 1. For QDH, SEM images show a needle morphology. The QDH crystals are very small in size compared to the QDMSO crystals, approximately 30 μm in length instead of 100 of μm for QDMSO, as shown from the SEM image analysis. The crystals do not seem to have a well-defined shape, and the different facets of the needle crystals, especially the capping faces, are not clearly visible. The not clearly visible facets of QDH could be a result of impurities during crystallization. Although the same starting material was used for the crystallization of QDMSO, the QDMSO crystals grew bigger, which could have minimized the effect of such impurities. It is also possible that the impurities were more easily incorporated into the growing QDMSO crystal, with less effect on the morphology. The badly defined facets of QDH could impact the experimental surface energy measurements for the QDH crystals. Due to the slow growth kinetic and low water solubility, suitable size crystals of QDH for single crystal indexing could not be obtained; hence, PXRD was used to characterize the crystals. Figure 2a shows the experimental PXRD pattern of the crystallized sample together with the simulated pattern of QDH as obtained from the Mercury software. Comparing the two, it can be seen that the intensity of certain peaks is enhanced in the experimental pattern. It has been reported in the literature that crystalline materials with largely exposed facets tend to orient in a particular direction during PXRD analysis on a horizontal sample holder; thus, the diffraction peaks corresponding to the lattice planes that are parallel to the sample holder itself are intensified.^45,46^ For QDH, the planes corresponding to the peaks that exhibited considerably higher intensity than the simulated pattern were identified and were found to be planes (020) and (300). The (020) plane is part of the {0*k*0} indices family; therefore, it indicates the presence of a dominant {010} facet on the crystals measured. Additionally, the (300) plane belongs to the {*h*00} indices family and confirms the presence of a large exposed {100} facet.

For QDMSO, SEM images show a thin, plate-like morphology. From the SEM images, three different facets can be distinguished: one large flat surface and two different side facets of a much smaller relative surface area. The PXRD data for these crystals (Figure 2b) show that some peaks have a greatly enhanced intensity compared to the other characteristic peaks for QDMSO, which were found to be for the (002), (004), (006), and (008) planes, all belonging to the {00*l*} indices family. This indicates a large exposure of the {002} facet for the particles produced in this study. Furthermore, single crystal face indexing for QDMSO was performed and confirmed the identity of the dominant flat surface to be the {002} facet.

The experimental data were compared with predicted morphologies of the two solvated structures using the attachment energy (AE) and the BFDH models. This first study allows one to verify if the dominant facets observed in the crystallized particles can be described computationally.

**Figure 3:**
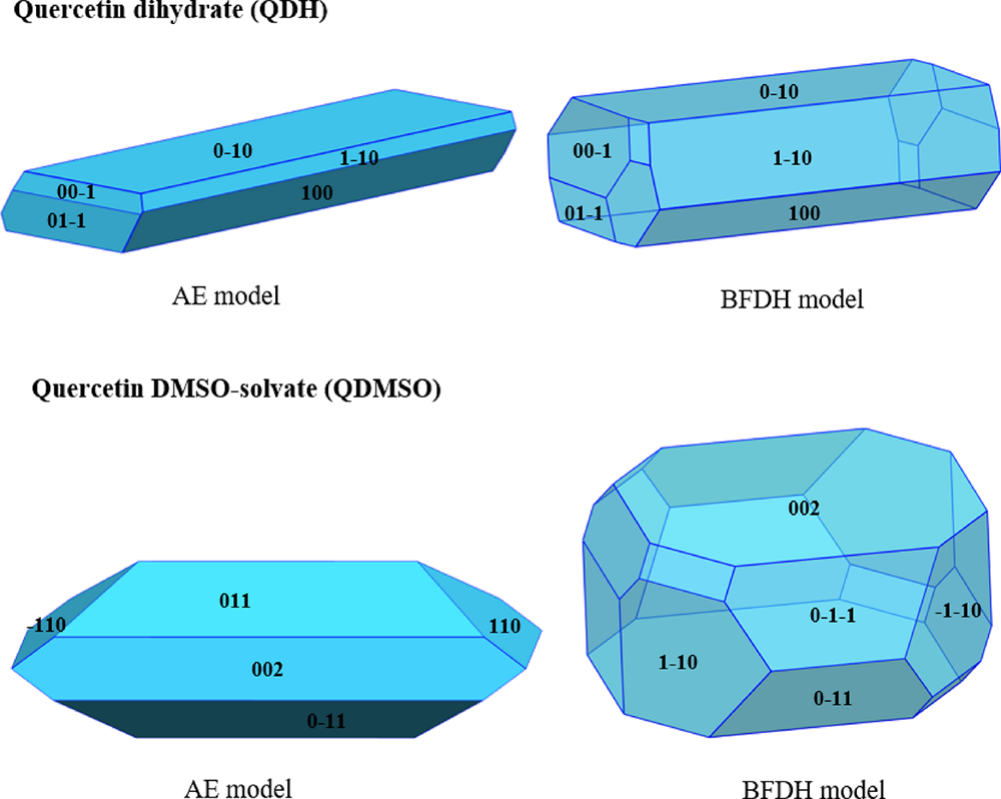
Attachment energy (AE) and BFDH model morphological predictions for QDH and QDMSO showing the major faces that are predicted by the models.

Figure 3 shows the AE and BFDH predicted morphologies for the quercetin dihydrate and the DMSO solvate.

For QDH, both models predict a needle morphology, with {010} facets being of highest morphological importance followed by {100} facets. This is in good agreement with the experimental data that indicated the {100} and {010} facets as dominant and that showed a needle-like morphology.

For QDMSO, a plate-like morphology is predicted by both models; however, the AE model predicts the {011} facets to be the most dominant followed by {002}, while the BFDH model shows the {002} to be the largest exposed face, with {011} being the second most dominant.

The crystals obtained experimentally present dominant {002} facets, indicating that the BFDH model’s morphological prediction is a better match to the experimental morphology for the QDMSO solvate.

The AE model assumes the growth of the crystals to take place in a vacuum and at a low driving force. As discussed later, the model predicts hydrogen bonds on the {002} surface to be very dominant, hence a higher attachment energy for {002} compared to {011} and therefore faster growth rate and lower morphological importance. However, it is observed that, experimentally, {002} is the most dominant facet and hence grows slower, probably due to strong interactions of the face with the polar crystallization solvent (DMSO and water) that reduces the growth rate of the surface.

The BFDH model does not account for the strong hydrogen bonds on the {002}, while the van der Waals forces of attraction, which are more prevalent on the {011} facet, are well accounted for. For molecules to form strong van der Waal forces, they have to pack closely together, so interplanar spacing is usually small when there are a lot of van der Waal forces. For hydrogen bonding, such a close packing is not required; thus, it is not well accounted for by the BFDH model. As a result, the BFDH model predicts a faster growth rate for the {011} facets and a higher morphological importance for the {002}. It should be noted that neither of the two models takes into consideration the effect of a solvent on crystal growth, which indeed has an important effect on the morphology.^43,47−49^

As the BFDH model gives a better match to the observed morphology, the BFDH morphology prediction will be shown in the following discussions; however, the attachment energy calculations are still used for the surface chemistry analysis.

### Surface Chemistry Analysis

The extrinsic synthons and the specific unsaturated interactions that contribute to the attachment energy and growth for each dominant facet of the QDH and QDMSO structures were calculated and characterized. The 10 strongest intermolecular synthons found in the lattice of QDH and QDMSO were considered for the analysis, and the contribution of each synthon to the growth of the facets was calculated. This information is summarized in Tables 1 and 2 for QDH and QDMSO, respectively. More detailed characterization for the six strongest synthons, which was also included in our previous publications, is shown in Table S3 in the Supporting Information (SI).^30,31^

**Table 1:**
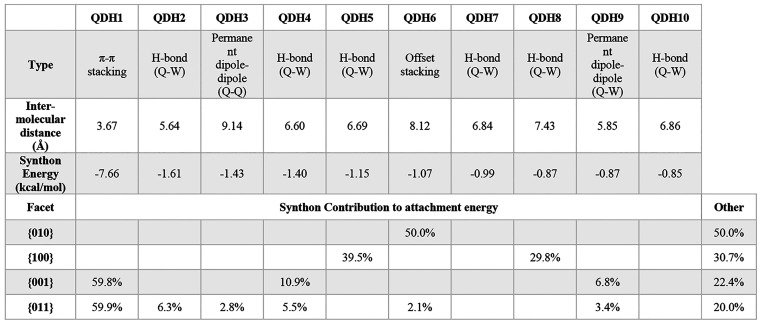
Synthon Contribution to the Attachment Energy and Growth of the Facets of QDH

**Table 2:**
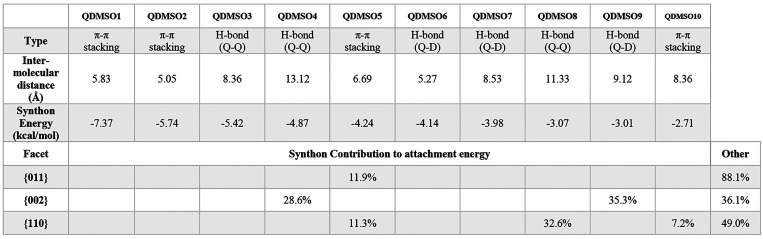
Synthon Contribution to the Attachment Energy and Growth of the Different Facets of QDMSO

#### Quercetin Dihydrate (QDH)

**Figure 4:**
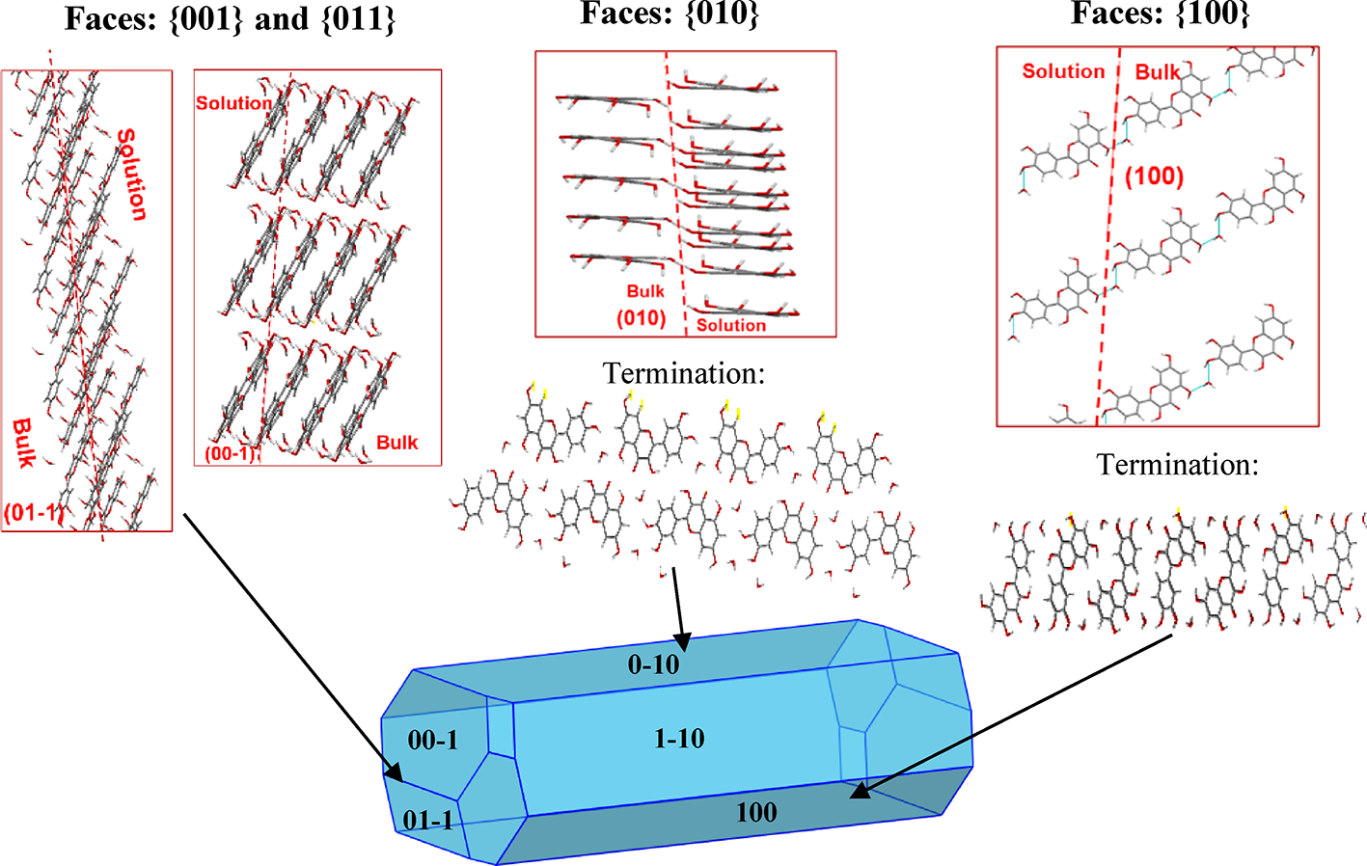
Surface chemistry analysis schematic for QDH showing the growth intermolecular interactions by which the {010}, {100}, {001}, and {011} habit planes of QDH grow. Light blue lines indicate hydrogen bonds.

For the most dominant facet {010}, it was found that none of the first five strongest synthons contribute to the attachment energy of this facet; instead, synthon QDH6, which is an offset stacking interaction between two quercetin molecules, is the main way of growth of this facet. The strength of that synthon was predicted to be seven times smaller than the strongest synthon in the lattice of QDH, which is consistent with the low attachment energy and growth rate of the facet. The contribution to the attachment energy of this facet comes mainly from the exposed −OH groups at the surface termination, which participate in this offset stacking of quercetin molecules. The aromatic hydrogens on the phenyl and pyrone rings also participate in the stacking interactions and contribute to the attachment energy of the facet. No hydrogen bonding was found to contribute to the growth of the {010} facet, as shown in Figure 4. The fact that facet {010} terminates with the −OH groups but does not grow via hydrogen bonds (not even involving water molecules) is a particularly interesting observation; this behavior could be due to the orientation of the quercetin molecules on the facet, which prevents the −OH groups from forming hydrogen bonds with other incoming quercetin or water molecules. Instead, stronger π–π stacking interactions could be preferentially formed on this facet. In general, this behavior could indicate that this facet has a nonpolar nature.

On the contrary, most contribution to the growth of facet {100} comes from synthons QDH5 and QDH8, which are quercetin–water hydrogen bonds. The surface termination shows the exposed oxygens on the hydroxyl groups of the pyrone ring of the quercetin molecule that are available to participate in hydrogen bonding with the water molecules from the solution. It is therefore expected that this family of facets would present a strong polar nature.

The needle capping facets {001} and {011} that were predicted to have the highest attachment energy and growth rate were found to have very similar surface chemistries. The growth direction of these facets is almost parallel to the π–π stacking of the quercetin molecules, the strongest synthon in the structure (QDH1), which was found to have the greatest contribution to their growth. At the same time, hydrogen bonding between the hydroxyl groups of the quercetin molecules and water molecules (e.g., QDH2 and QDH6 for {011} and QDH4 for both {011} and {001}) was also found to contribute to their growth. The quercetin molecules were found to pack more closely along those facets, which favor the faster growth. Because both π–π stacking interaction and hydrogen bonds contribute to the growth of those facets, it is predicted that these are highly energetic facets with a capability of forming both polar and nonpolar interactions.

#### Quercetin DMSO-Solvate (QDMSO)

For QDMSO, the attachment energy model predicted the facet {011} to be of the highest morphological importance followed by the facet {002}, which was in fact shown earlier to be the dominant facet for the QDMSO sample prepared experimentally. It was found that for the facet group {011}, the synthon of highest energy that contributes to growth is QDMSO5, which is a nonpolar π–π stacking interaction between two quercetin molecules. In this particular synthon, the aromatic carbons and aromatic hydrogens from the pyrone and phenyl rings of the neighboring quercetin molecules are forming these π–π stacking interactions. The facet termination prediction confirms this by showing the phenyl and pyrone rings exposed at the surface. It is, hence, hypothesized that the {011} facet could have a nonpolar nature.

**Figure 5:**
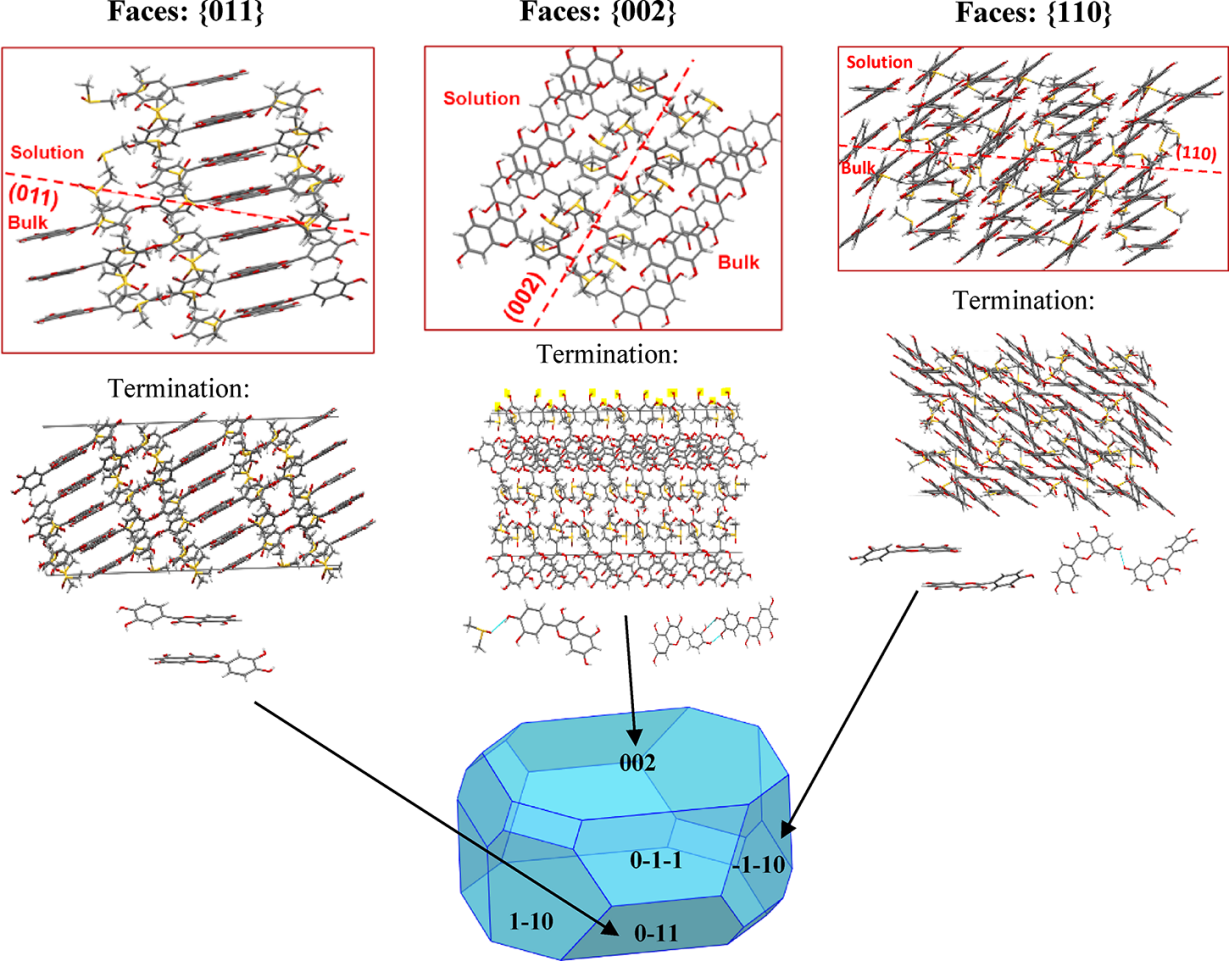
Surface chemistry analysis schematic for QDMSO showing the growth intermolecular interactions by which the {002}, {011}, and {110} habit planes of QDMSO grow. Light blue lines indicate hydrogen bonds.

On the other hand, facet {002} grows mainly from synthons QDMSO4 and QDMSO9, which are both hydrogen bonds. QDMSO4 is a double hydrogen bonding interaction between the hydroxyl groups of two quercetin molecules, whereas QDMSO9 is a hydrogen bond between a quercetin and a DMSO molecule. As seen in Figure 5, the hydroxyl groups, which could participate in these hydrogen bonds, are exposed at the facet termination. Because the exposed groups can form hydrophilic interactions with polar molecules like DMSO or water, it is suggested that {002} should have a polar nature. Although the attachment energy model predicts the {002} facets to be less dominant than {011}, experimentally, the {002}facets appear to have a much larger relative area than the {011} facets. This could be due to the fact that water was present in the solvent mixture used to crystallize the QDMSO particles. In fact, water is capable of forming hydrogen bonds on this facet, competing with quercetin and DMSO molecules. This competition would slow down the growth in the direction perpendicular to the {002} facet, resulting in this surface becoming dominant. The {110} facet, which appears on both the AE and BFDH models’ morphological predictions although it is not a dominant facet, was found to grow by a combination of nonpolar π–π stacking interactions, QDMSO5 and QDMSO10, as well as a weaker hydrogen bond between the hydroxyl groups of two quercetin molecules, QDMSO8. As shown in Table 3 below, the {110} facet is a surface of higher attachment energy, which is capable of forming both polar and nonpolar interactions. It is therefore predicted that the polarity of this facet will be lower than that of {002} but higher than that of {011}.

**Table 3:** Slice, Attachment, and Surface Energies of the Most Important Facets as Predicted by the Attachment Energy Rule

quercetin dihydrate (QDH)				
facet (hkl)	slice energy (kcal/mol)	attachment energy (kcal/mol)	surface energy (mJ/m^2^)	ε_hkl_
{010}	–13.1	–1.0	13.9	93.1%
{100}	–11.9	–2.2	27.8	84.5%
{001}	–5.0	–9.1	34.7	35.2%
{011}	–5.0	–9.2	34.7	35.2%

quercetin DMSO solvate (QDMSO)				
face (hkl)	slice energy (kcal/mol)	attachment energy (kcal/mol)	surface energy (mJ/m^2^)	ε_hkl_
{011}	–26.7	–4.00	20.8	92.0%
{002}	–25.1	–2.3	13.9	86.3%
{110}	–24.7	–4.3	13.9	85.1%

Overall, the surface chemistry analysis demonstrates that different solid forms of the same substance can have very different surface properties. The most morphologically important facets on the two quercetin solvate structures are shown to have a very different chemical nature owing to the different synthons that contribute to their growth and the different functional groups exposed. Their different surface properties are expected to affect their performance in downstream operations during processing, as well as their performance in formulations. Furthermore, the two solid forms demonstrated surface anisotropy, with different surface chemistry for their different facets. This can allow engineering the shape of the crystal, through the choice of solvents or crystallization and growth conditions, to manipulate the relative areas of the different facets and therefore change the overall surface properties of the solid form.

The attachment energy model also allowed for the calculation of slice energies and attachment energies for specific facets experimentally observed for the two quercetin solvates, as well as the anisotropy factors, as shown in Table 3.

The values for the surface energies of each facet of the QDH indicate a clear anisotropy for this crystal structure, as the dominant {010} facet presents a considerably lower value compared to the other families of facets. The QDMSO also shows different values of facet specific surface energies, which indicate surface anisotropy also for this crystal structure.

It is worth noticing that the overall surface nature of the particles will depend not only on the chemical properties of each facet but also on the amount of the total crystal area occupied by each facet, which is basically the crystal morphology.

### Bulk Contact Angle Measurements and Wettability

The wettability of compressed discs of QDH and QDMSO was assessed by measuring the contact angle of water droplets on their surface to evaluate how these crystals interact with the polar solvent and to assess their overall surface polarity. Facet specific water contact angle measurements were not possible due to the fact that crystals of suitable size for QDH or QDMSO could not be obtained. This technique measures a single parameter over all sites of a compressed powder surface, and the angle measured is an average depending on the relative area of the different facets present on the surface of the compressed disc. Thus, it does not give a complete picture of the surface anisotropy of a crystal. However, the average hydrophobicity/hydrophilicity of the two crystal structures tested can be compared and can be related to the surface chemistry of the most dominant facets in each form. The results are shown in Table 4.

**Table 4:** Water Contact Angle Measurements for QDH and QDMSO

QDH water contact angle measurement	48.0 ± 3.2°
QDMSO water contact angle measurement	38.8 ± 1.1°

The lower the water contact angle is, the more hydrophilic are the particles because the liquid has a higher tendency to spread on the disc surface. The results show that QDMSO is more hydrophilic than QDH. It was earlier shown that the most dominant facet in QDH was {010}, which grows by quercetin–quercetin interactions, and although −OH groups are present at the termination, no hydrogen bonds were observed to form among solute molecules on those facets, as seen in Figure 4 for the growth interactions of facet {010}. It can then be assumed that the attachment of water molecules on that facet is not as favorable as on the {002} surface of QDMSO, which was predicted to be polar as it grows by quercetin–quercetin hydrogen bonding due to the exposed −OH groups. Hence, it is reasonable to assume that this facet could easily form hydrogen bonds also with small molecules such as water. Therefore, it can be assumed that, for QDMSO, the very large relative area of {002} surfaces dominates the total surface area of the crystal; thus, the surface properties of the particle are dominated by the nature of this polar facet.

In QDH, the most dominant {010} surfaces are nonpolar, which should grant an overall hydrophobic surface behavior; however, a contribution from the more polar {100} surfaces should also be expected. Nevertheless, the larger water contact angle measured for the QDH disc means that, overall, the surface behavior of QDH is more hydrophobic compared to that of QDMSO owing to the large contribution of the hydrophobic {010} facets in QDH.

Because the two solid forms are anisotropic, the relative areas of the different facets should affect the value of the water contact angle measured, as the overall surface polarity would be determined by the contribution of all facets based on their relative areas. The water contact angle on QDH was previously measured using the exact same instrument of this work, and a value of 59.4 ± 0.4° was obtained.^50^ However, no information was given on the crystallinity, size, and shape distribution and crystal form for this measurement. All these parameters can affect contact angle measurements.

### Inverse Gas Chromatography (IGC)

**Figure 6:**
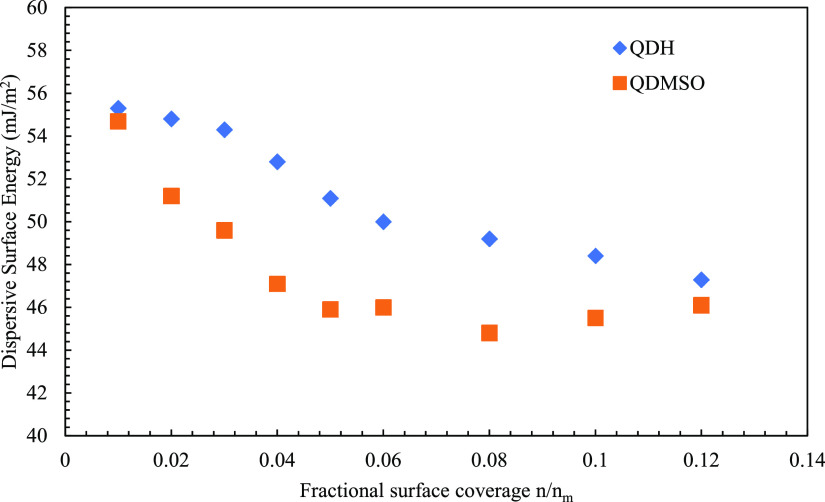
Overall dispersive surface energy as a function of surface coverage for QDH and QDMSO.

The contact angle measurements give a quantitative measurement for the bulk polarity of the two solvates studied, but it is not facet specific. IGC goes one step ahead because it can be used to evaluate the surface energy heterogeneity profile of the substances under study and to assess more precisely the surface chemistry anisotropy. IGC data give the relationship of the dispersive component of the surface energy at different surface coverages of alkane probe molecules. Because different crystal facets have different adsorption energies, it is expected that, for a heterogeneous material, the surface energy will decrease with increasing surface coverage as, at a lower surface coverage, the more energetic sites will interact with the alkane probes first. As the surface coverage increases, the interaction strength between the probe molecules and the less energetic sites will be weaker. All measurements shown here were carried out at 35 °C and 10% relative humidity (RH). To confirm that the two solvates were stable at these conditions, the specific surface area (SSA) of the crystals was measured at different RH using an octane isotherm Brunauer–Emmett–Teller (BET) method. As desolvation processes are often associated to changes in the shape of a crystal, SSA measurements at different RH values can give an indication of the presence of solid phase transition (Supporting Information Tables S1 and S2 and Figure S1). To validate the BET SSA measurements, the crystal size distribution for QDH and QDMSO was also measured using image analysis (Morphologi G3). The methodology and results for these are shown in Supporting Information Figures S2–S4. The IGC data for the two quercetin solvates are shown in Figure 6 for the dispersive component of the surface energy.

It can be observed from the IGC data that both QDH and QDMSO show surface energy heterogeneity; i.e., the surface energy changes as a function of the surface coverage. This could be attributed to the crystalline anisotropy exhibited by both the molecules and the resulting differences in the surface chemistry of the different facets. At low fractional surface coverages, the probe molecules will preferentially interact with the most energetic sites, with which they can form the strongest nonpolar interactions. With the increase in the fractional coverage, the probe molecules will interact with the less energetic sites and overall cover the changing surface energy landscape of the material. This explains the decreasing trend of the overall dispersive surface energy as depicted in Figure 6.^3−5,51^ Thus, the anisotropy in the QDH and QDMSO crystals was confirmed from the surface energy heterogeneity data, which are also in line with the modeling calculations that predicted facets of both solvates to have different surface chemistries and polarities.

When comparing the two crystal structures, the measured energy range for QDMSO is higher, from 54.7 mJ m^–2^ at a surface coverage of 0.01 to 44.8 mJ m^–2^ at a surface coverage of 0.08. Meanwhile, QDH only changes from 55.3 to 47.3 mJ m^–2^ for values of surface coverages between 0.01 and 0.12. This suggests that the anisotropy in surface chemistry and perhaps the difference in polar nature among facets are greater for QDMSO than for QDH.

It is worth comparing the IGC data with the surface energies calculated with the attachment energy model (Table 3): both techniques show that the QDH and QDMSO are anisotropic, but IGC measurements indicate a higher anisotropy for the QDMSO as opposed to the modeling results. This smaller variability in the QDH dispersive surface energy compared to the modeling results could be partly attributed to the fact that these crystals have poorly defined facets, which might be contributing less to the energies measured by the IGC, as compared to the much more well-defined facets of the QDMSO.

Although the surface energy range for QDMSO is greater, the actual value seems to reach a plateau after a surface coverage of 0.06, while the surface energy for QDH keeps decreasing even at higher surface coverages. To explain this phenomenon, the following two factors need to be considered: (1) the predicted polar nature of the {002} facets, which are expected to have a lower interaction energy with the alkane probe molecules than the nonpolar facets {011} and {110}, and (2) the higher relative surface area of the {002} facets in the QDMSO crystals compared to the other facets (SEM images indicated that this family of facets accounts for approximately 95% of the total surface area of the QDMSO crystals).

Given these premises, the nonpolar {011} and {110} facets are likely the first to form interactions with the probe molecules at higher values of the surface energy. Then the probe molecules start to cover the large {002} facets at a constant value of dispersive surface energy, which could be assumed to be the specific one of the alkane molecules with the {002} facets.

When the magnitude of the dispersive surface energy is compared for the two structures, it can be seen that, at all surface coverages, the energy for QDH is higher compared to that for QDMSO. Because the dispersive component of surface energy is a measure of the van der Waals interactions, it is suggested that, overall, the facets of QDH are more nonpolar compared to QDMSO. This is in agreement with the modeling results that showed that the most dominant facet of QDH {010} is nonpolar as it grows mainly by offset π–π stacking interactions, while the largest facet for QDMSO {002} is polar because it grows by quercetin–quercetin and quercetin–DMSO hydrogen bonds. It also agrees with the contact angle measurements, which showed that QDH is more hydrophobic than QDMSO. Moreover, some of the differences in the results comparing the magnitude of the facet specific surface energies of the single crystal with the weighted average surface energies are in the acceptable range as shown from some of our previously published studies with different crystalline materials.^52−54^

Overall, the IGC and contact angle measurements show a reasonable agreement with the surface chemistry prediction from the modeling work, demonstrating that both solvates are anisotropic, exhibiting a surface energy heterogeneity for the dispersive component, and that the QDH has overall a more hydrophobic nature compared to the QDMSO.

## Conclusions

A molecular modeling analysis has been conducted on two solid forms of quercetin to rationalize the surface properties of this material through the study of the extrinsic synthons and morphologies. The modeling calculations were then compared to experimental work including IGC and contact angle measurements.

Via synthonic modeling, the attachment energies and surface anisotropy factor for the two quercetin solid forms were calculated, along with the predicted morphologies. Those were compared to SEM images of the crystals and PXRD data. The surface chemistry analysis confirmed the anisotropy of the two solid forms and helped in the characterization of the hydrophobicity of their surfaces.

For QDH, the {010} facets were predicted to be hydrophobic as they grow mainly by a nonpolar offset quercetin–quercetin stacking interactions, while the {100} facets were expected to be hydrophilic as the main growth interaction is a polar quercetin–water hydrogen bond. For QDMSO, the dominant facet {002} grows by a strong polar quercetin–quercetin hydrogen bonding interaction, while the second most dominant facet {011} grows by nonpolar π–π stacking interactions.

The contact angle measurements showed that the QDMSO form has a greater overall surface hydrophilicity compared to QDH. The IGC data demonstrated surface energy heterogeneity for both structures, as the surface energy changed as a function of surface coverage. The data showed a greater heterogeneity in the properties of the facets of QDMSO, as it spanned a greater range of surface energies. The dispersive component of the surface energy for QDH was found to be greater than QDMSO at all surface coverages, which indicated a greater overall hydrophobicity for QDH.

In general, the modeling results combined with the experimental findings demonstrated facet-specific anisotropy in the surface properties of the different quercetin solid forms studied. This includes heterogeneous surface energy along the different facets, and different hydrophobicity and polar nature of facets. It is worth noticing that particle anisotropy is related not only to the chemical nature of each facet present on the crystal but also to its morphology.

This information is vital to know when designing solid forms for a particular application. The approach used in this work can be applied to design particles with the optimal crystal structure and morphology and to guide the choice of crystallization solvent and other crystallization parameters such as solvent composition and supersaturation that will affect crystal properties.

## Supporting Information Available

The methodology and the experimental results for the BET analysis of quercetin dihydrate and quercetin DMSO crystals at different RH values, a summary of the six strongest bulk intermolecular synthons for QDMSO and QDH and their properties, and the results of the particle size measurements conducted with the Malvern Morphologi G3 equipment (PDF)
