# Supplementary material for: Relating Crystal Structure to Surface Properties: A Study on Quercetin Solid Forms
Source: Cryst Growth Des. 2022 Sep 19;22(10):6103–13. doi: 10.1021/acs.cgd.2c00707 (PMC9542717; doi:10.1021/acs.cgd.2c00707)
Supplement: Supplementary file 1 — cg2c00707_si_001.pdf [file cg2c00707_si_001.pdf]

SUPPORTING INFORMATION FOR: RELATING CRYSTAL STRUCTURE TO SURFACE PROPERTIES: A STUDY ON QUERCETIN SOLID FORMS

Panayiotis Klitou[1], Ian Rosbottom[2], Vikram Karde[2], Jerry Y Y Heng[2], Elena Simone[3,1]*

[1]School of Food Science and Nutrition, Food Colloids and Bioprocessing Group, University of Leeds, Woodhouse Ln., Woodhouse, Leeds LS2 9JT, United Kingdom

[2] Department of Chemical Engineering, Imperial College London, Imperial College Rd, South Kensington, London SW7 2AZ, United Kingdom

[3] Department of Applied Science and Technology, Politecnico di Torino, Corso Duca degli Abruzzi, 24, 10129 Torino TO, Italy

*Corresponding author: elena.simone@polito.it

Brunauer-Emmett-Teller (BET) analysis of Quercetin dihydrate and Quercetin DMSO crystals using Inverse Gas Chromatography at different RH

Method

BET using IGC

The Quercetin crystals were studied for their BET using Inverse Gas Chromatography, IGC surface energy analyzer (IGC *SEA*, SMS, UK). Due to the difference in the specific surface areas of the 2 samples different amounts of samples were used for the analysis. About 25 mg of the Quercetin dihydrate and 115 mg of Quercetin DMSO sample was packed into a salinized glass column (internal diameter = 4 mm) and plugged with salinized glass wool on both the ends. A jolting voltameter (Surface Measurement Systems, London, UK) was used to provide mechanical tapping to the sample in order to remove the voids in the packed sample bed. The packed sample column was analyzed using octane as a solvent at 10% RH , 30% RH and 50% RH conditions at a temperature of 35˚C. Prior to BET measurement, the sample column was conditioned at the same RH and temperature conditions for a period of 2 h with Helium as a carrier gas at 10 ml · min^{-1} carrier gas flow. Methane was used as a reference gas to determine the dead volume.

Results

Table S1. Specific surface area using octane isotherm in IGC

Quercetin dihydrate

RH (%)	BET SSA (m^2/g)
10	31.4

30	31.1
50	31.4

Table S2. Specific surface area using octane isotherm in IGC

Quercetin DMSO

RH (%)	BET SSA (m^2/g)
10	6.3
30	6.2
50	6.4

The BET SSA values for both QDH and QDMSO do not change considerably as a function of the RH. As de-solvation processes are often associated to changes in surface features or shape of a crystal, the constant SSA values indicate that the two structures do not undergo any de-solvation process within the RH range tested. As further confirmation of this hypothesis Figure S1 shows almost identical octane retention behavior for the QDH column at two different level of RH.

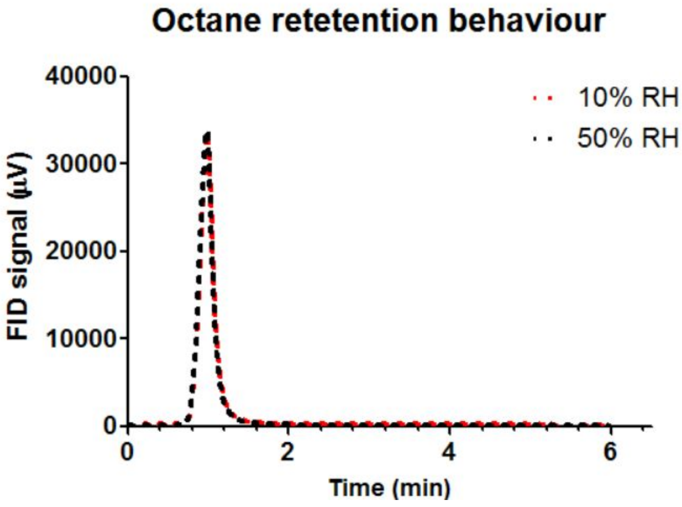

Figure S1: Retention behavior of Octane probe at equal coverages for 10% and 50% RH exposed QDH sample.

Table S3. Summary of the six strongest bulk intermolecular synthons for QDMSO and QDH and their properties. [1]

	QDMSO Intermolecular Interactions						QDH Intermolecular Interactions					
**Main synthons**	**QDMSO1**	**QDMSO2**	**QDMSO3**	**QDMSO4**	**QDMSO5**	**QDMSO6**	**QDH1**	**QDH2**	**QDH3**	**QDH4**	**QDH5**	**QDH6**
Type	π-π stacking	π-π stacking	H-bond (Q-Q)	H-bond (Q-Q)	π-π stacking	H-bond (Q-D)	π-π stacking	H-bond (Q-W)	Permanent dipole-dipole	H-bond (Q-W)	H-bond (Q-W)	Offset stacking
Intermolecular distance (Å)	5.83	5.05	8.36	13.12	6.69	5.27	3.67	5.64	9.14	6.60	6.69	8.12
Synthon Energy (kcal/mol)	-7.37	-5.74	-5.42	-4.87	-4.24	-4.14	-7.66	-1.61	-1.43	-1.40	-1.15	-1.07
% contribution of aromatic rings to synthon	55.8	74.4	54.4	21.3	63.6	33.3	66.7	11.3	56.0	5.4	17.2	33.0
% contribution of hydroxyl groups to synthon	22.2	15.2	34.5	83.1	34.3	45.2	23.0	72.7	27.1	91.8	71.8	49.0
% contribution	22.0	10.4	11.1	−4.4	2.0	21.5	10.3	15.7	16.9	2.8	11.0	18.1

of carbonyl bond to synthon												

Morphologi G3 size measurements

Morphologi G3 is a particle characterization technique using imaging and data analysis, which can measure the particle size and shape distribution of extremely large number of crystals. The technique measures the area of a 2D image of the particle which can be used to calculate a circle equivalent diameter distribution.

The Circle Equivalent (CE) diameters for QDH and QDMSO are shown in the figures below.

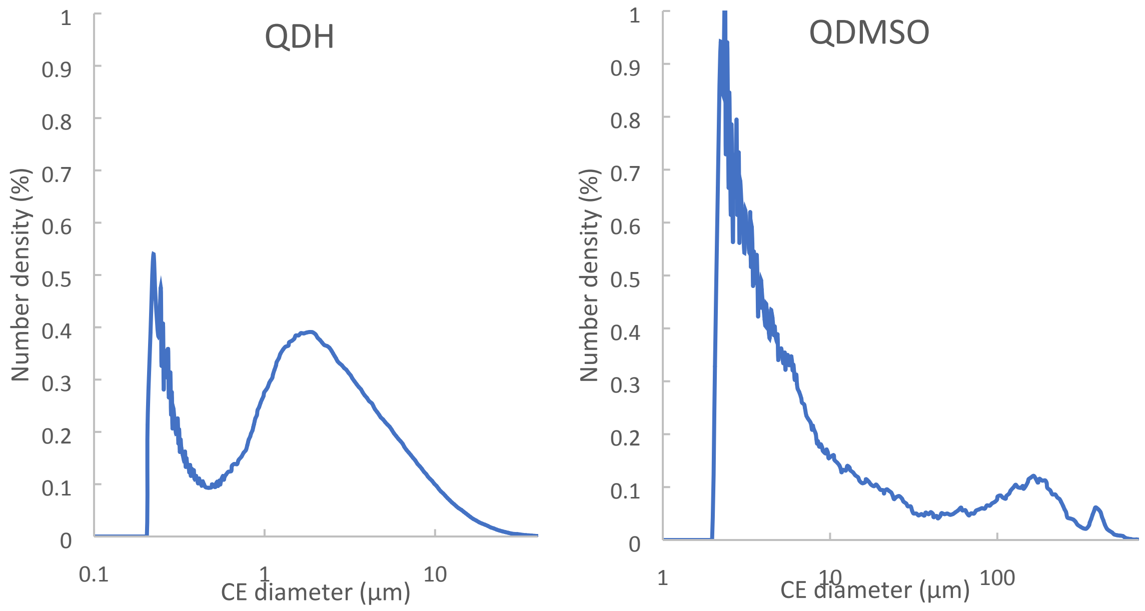

Figure S2. Circle Equivalent (CE) diameters for QDH and QDMSO

For the QDH crystal specific surface area (SSA) approximation from the Morphologi G3 measurements, certain assumptions had to be made:

1. The QDH crystals have rectangular cuboid shape.
2. The aspect ratio of the crystals (width/length) was 0.56 for the whole population. This was the peak aspect ratio measured by the technique.

3. The thickness of the crystals is 1/3 of the width. This assumption was made based on measurements taken from SEM images of QDH which showed that usually the thickness was 1/3 of the width.

The density of QDH (1600.24 kg/m^3) was used to calculate the mass based on the predicted volume.

The predicted SSA for QDH was 10.18 m^2/g. This is approximately 3 times smaller than the SSA measured from the BET octane isotherm method before the IGC. However, it is expected that the prediction from the Morphologi G3 data is greatly underestimated due to the significant degree of agglomeration of the QDH crystals which the software recognizes as a single particle. This means that the agglomerated sides of the crystals would not contribute to the predicted SSA. An example of agglomerated QDH crystals is shown below.

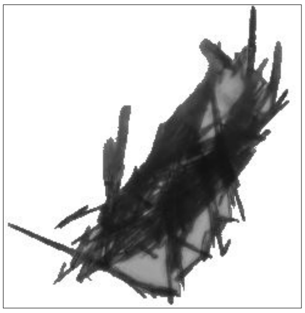

Figure S3. Agglomerated QDH crystals shown on Morphologi G3 size measurement tool.

For the QDMSO SSA prediction, different assumptions were made:

1. The shape of the QDMSO crystals are circular plates.
2. The thickness of the plates is 0.026 times the circle equivalent diameter of the circular surface.

The SSA was calculated to be 11.39 m2/g, where as the BET measured one was 6.3 m2/g. This overestimation is likely to be due to the overlapping agglomeration of the QDMSO crystals as seen from the software imaging. Example image is shown below.

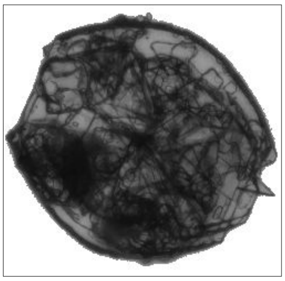

Figure S4. Agglomerated QDMSO crystals shown on Morphologi G3 size measurement tool.

REFERENCES

[1] P. Klitou, C. M. Pask, L. Onoufriadi, I. Rosbottom, and E. Simone, "Solid-State Characterization and Role of Solvent Molecules on the Crystal Structure, Packing, and Physiochemical Properties of Different Quercetin Solvates," *Cryst. Growth Des.*, vol. 20, no. 10, pp. 6573–6584, Oct. 2020, doi: 10.1021/acs.cgd.0c00751.
